# Computational Investigation on the Photoacoustics of Malaria Infected Red Blood Cells

**DOI:** 10.1371/journal.pone.0051774

**Published:** 2012-12-14

**Authors:** Ratan K. Saha, Subhajit Karmakar, Madhusudan Roy

**Affiliations:** 1 Ratan K Saha Applied Material Science Division, Saha Institute of Nuclear Physics, Kolkata, West Bengal, India; 2 Subhajit Karmakar Applied Nuclear Physics Division, Saha Institute of Nuclear Physics, Kolkata, West Bengal, India; 3 Madhusudan Roy Applied Material Science Division, Saha Institute of Nuclear Physics, Kolkata, West Bengal, India; Liverpool School of Tropical Medicine, United Kingdom

## Abstract

A computer simulation study on the possibility of using photoacoustic (PA) technique to differentiate intraerythrocytic stages of malarial parasite is reported. This parasite during its development substantially converts hemoglobin into hemozoin. This conversion is expected to alter the cellular absorption leading to changes in the PA emission of a red blood cell (RBC) at certain incident optical wavelengths. The PA signals from blood samples corresponding to ring, trophozoite and schizont stages were computed and compared with that of normal blood. A Monte Carlo algorithm was implemented generating random locations of RBCs in 3D to simulate blood samples. The average PA amplitude for wide bandwidth signals decreases for 434 nm incident radiation, but increases for 700 nm as the parasite matures. The spectral power at 7.5 MHz for the blood sample at the schizont stage compared to the normal blood is nearly reduced by 6 dB and enhanced by 22 dB at those incident wavelengths, respectively. Bandlimited signals for transducers of 15 and 50 MHz center frequencies were studied and found to exhibit similar characteristics. The presence of hemozoin inside the cells was examined and an excellent estimation was made. The simulation results suggest that intraerythrocytic stages of malarial parasite may be assessed using the PA technique.

## Introduction

It is indeed alarming that malaria continues to be a major threat to human life in tropical and sub-tropical regions of our planet. Approximately, 225 million people worldwide suffer from malaria per year and the disease kills a child every 30 seconds in endemic areas. These deaths are largely due to the infection of red blood cells (RBCs) by the parasite known as *Plasmodium falciparum* [1]. There exist also some other species (e.g. *Plasmodium vivax*, *Plasmodium ovale* and *Plasmodium malariae*) which can infect RBCs leading to a non-fatal mild disease [1]. In fact, the existence of such parasites inside the RBCs of patients having malaria was first observed microscopically by Charles L. A. Laveran in 1880 [1]. The early stage of the parasite within a host RBC is referred to as ring stage because of its ring type appearance under the microscope. The intermediate growth period is known as trophozoite. The ring-form morphology is disappeared during this period. The mature stage of the parasite is called as schizont stage. Several merozoites are produced during this stage due to asexual amplification. When a schizont ruptures, these merozoites are released into the blood stream and these in turn infect more number of RBCs. In 1898, Ronald Ross proved that the parasites complete their life-cycle in the gastrointestinal tract of the *Anopheles* mosquito and established that it is a mosquito-borne disease.

An accurate, rapid, simple and low cost diagnostic system is required for the detection of malarial infection in its early stage followed by timely administration of antimalarial drugs to combat epidemic break outs of malaria in tropical and sub-tropical regions. Generally, high power microscopic examination of Giemsa-stained thick blood smears is employed to diagnose malaria mostly in laboratory setting as a gold standard till today [2]. However, it is a laborious procedure and moreover, the accuracy of this test depends significantly on the efficiency of the technician. Thus, it finds limited applications for on-field diagnosis of malaria. Other avenues such as the fluorescent microscopy and the molecular techniques have also been explored [2]. These techniques are in general complex, expensive and time consuming. Such drawbacks have not facilitated each of them to evolve as a popular practical diagnostic tool. Besides, malaria antigen detection based commercial equipments are also currently available for rapid diagnosis [3]. Nevertheless, these tests are expensive and less sensitive than the first method discussed above.

Malaria diagnosis through magneto-optical route has been investigated by Newman et al. [4]. It detects basically the presence of hemozoin (Hmz) crystals (released by the infected cells due to hemolysis) in blood serum by measuring optical modulation signals under the action of an external magnetic field. The presence of Hmz crystals in blood is an indication of malarial infection and the amount of Hmz crystals is somewhat related to the level of parasitemia. Initial results confirmed that this technique can match or exceed existing diagnostic techniques. A small preliminary clinical trial on 13 patients was conducted to validate the approach and satisfactory results were obtained. Recently, another group exploited non-linear optical effect generating third harmonic to image malaria infected RBCs [5]. Essentially, Hmz crystals, contained by the malaria infected cells, can produce very strong third harmonic signal. They measured that the signal strength for uninfected RBCs was very weak (on the order of background noise). The signal to noise ratio for Hmz crystals was about 1000∶1, allowing precise localization of the crystals in the image. Park et al. used tomographic phase microscopy (TPM) for quantitative estimation of refractive index distribution in malaria parasite invaded human RBCs [6]. The locations of Hmz crystals can clearly be identified from the TPM images. They also measured membrane fluctuations of infected RBCs employing the diffraction phase microscopy and demonstrated that spatially averaged membrane stiffness at the schizont stage was up to an order of magnitude higher than that for healthy RBCs.

In addition to that, a number of studies were directed to understand the biochemistry of malaria pigment (i.e. Hmz) [7], [8], [9], [10], [11], [12]. The malarial parasite during its intraerythrocytic development digests considerable amount of hemoglobin (Hb) and produces Hmz (insoluble polymerized forms of haem). Note that Hmz is structurally identical to 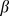-hematin, which is made of dimers of hematin molecules [7]. Proper understanding of Hmz crystal structure is crucially important to elucidate how antimalarial drugs (e.g. chloroquine) interact with it and inhibit the formation of 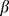-hematin. Several experimental techniques (e.g. microscopy, spectroscopy, X-ray diffraction, Mössbauer etc.) were employed to assess the efficacy of antimalarial drugs. The photoacoustic (PA) technique was also used to achieve this end [13], [14], [15]. PA is a non-invasive and hybrid modality. It probes optical and thermoelastic properties of a tissue by detecting pressure transients produced by the absorption of light [16], [17]. This technique has found its usage in characterization (e.g. blood oxygen saturation level estimation) and visualization (e.g. imaging of melanin in tissue) of bio-medical samples, as also in calorimetric studies [18]. It can probe deep tissue regions because pressure waves are detected in the receiving end whose scattering is two to three orders of magnitude less than that of light and thus, can travel longer distance in tissue. Based on the PA spectra, Balasubramanian and his group [13], [14] showed that chloroquine-treated parasites reveal *in situ* interaction between drug and ferriprotoporphyrin. Moreover, chloroquine-resistant parasites, readily distinguishable by this method, appear to degrade Hb only partially. In this work, they used mechanically chopped light beam to illuminate samples and captured the PA signals in the audiofrequency range (10–10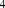 CPS, CPS stands for cycles per second). Nevertheless, no analysis was made in the diagnostic (2–10 MHz) and high frequency (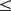
50 MHz) ultrasound regime. Recently, Sanson et al. [15] employed the PA spectroscopy to characterize 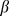-hematin. They showed that the PA absorption spectrum mimics the curve which is generated by optical spectroscopy. It was also reported that the prolonged irradiation dramatically alters the physical and optical properties of the 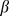-hematin resulting in increased absorption at shorter wavelengths. However, to the best of our knowledge, the PA signal properties have not been investigated as a function of parasitic stage.

A theoretical model has been developed recently to describe the PA field generated by a collection of spherical fluid absorbers [19], [20], [21]. It uses a frequency domain approach to express the single particle field [22] and that of many particles has been obtained by adding the fields emitted by the individual absorbers. It enables us to investigate the effect of spatial organization, size dispersity, bio-physical and bio-chemical properties of the absorbers on the PA signals. This model provided results consistent with the experimental findings. For example, this theory predicted that the PA signal amplitude would increase monotonically with increasing RBC aggregation [19] and that was verified through *in vitro* experiments using human and porcine RBCs [23]. The effect of RBC oxygen saturation on the PA signal was studied in depth employing this theoretical framework and meaningful results were obtained for various incident optical radiations and ultrasound receiver bandwidths of interest [20], [21].

The goal of this paper is to examine the potential of the PA technique to detect malarial infection in blood. The PA method can sense signals from deep tissue regions and thus, it may provide a means to monitor such an infection non-invasively. The PA signals from various blood samples were simulated using the above mentioned theoretical model. The molar extinction coefficients of Hb and Hmz are different at certain spectral bands. Hence, it is expected that cellular absorption coefficients for infected RBCs would differ from that of healthy RBCs. This in turn should lead to an appreciable difference between the PA signals obtained from blood samples with infected and normal cells. In this work, a Monte Carlo algorithm was implemented to construct spatial organizations of cells in the illuminated 3D region of interest (ROI). Such configurations were used to simulate the PA radio frequency (RF) signals corresponding to three intraerythrocytic stages of malarial parasite (namely, ring, trophozoite and schizont) for 434 and 700 nm incident laser radiations, respectively. For comparison, signals from normal cells were also computed. The simulated RF lines were then analyzed to obtain time domain and ultrasound spectral features. Our computer simulation results demonstrate that the PA technique has the ability to distinguish between various stages of infection using appropriate optical wavelength for the input irradiation. Further, it has been shown that the amount of Hmz present in a cell can also be estimated using the RF lines generated at those light wavelengths.

The paper is organized as follows. The theoretical framework and the simulation methods are described in the next section. The simulation results are presented in section 3. Discussions on the model and results are provided in section 4. The summary and conclusions are given in section 5.

**Figure 1:**
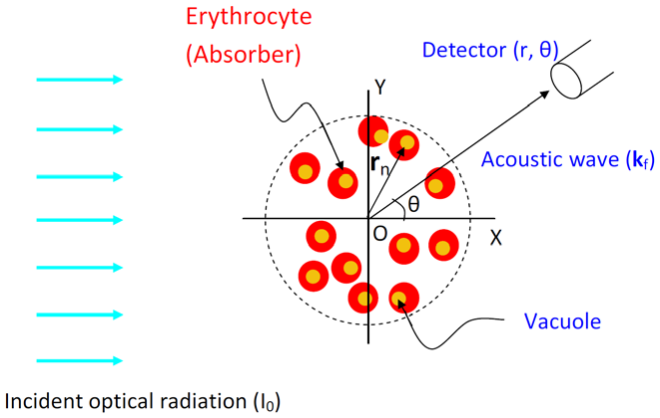
2D view of the PA geometry.

## Materials and Methods

### Theoretical Framework

While the theoretical framework has been presented earlier in detail (see [19], [20], [21]), however, for ready reference, the essentials are given below.

**Figure 2:**
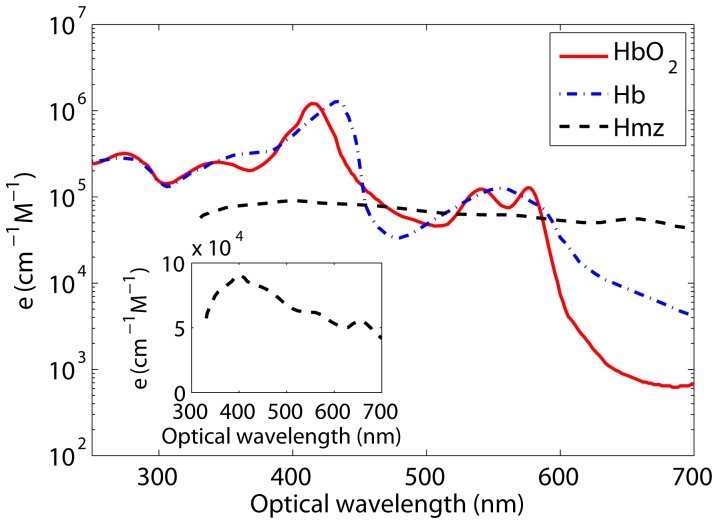
Plots of molar extinction coefficient for oxy-hemoglobin (HbO_2_), hemoglobin (Hb, commonly referred to as deoxy-hemoglobin) and hemozoin (Hmz) molecules. The curve for Hmz is further shown in the inset to highlight its characteristic features. Molar extinction coefficients at different optical wavelengths for the Hmz were estimated from curve a, left panel of Fig. 1 of [9].

#### Modeling of the PA field by spherical absorbers

The time dependent wave equation for the pressure field originating due to absorption of light under the condition of thermal confinement (i.e. heat conduction takes place after the acoustic pulse is launched) can be expressed as [22],
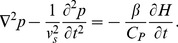
(1)


Here, 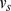, 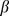 and 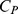 are the speed of sound, thermal expansion coefficient and isobaric specific heat for the absorbing region, respectively; 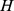 is the amount of heat energy deposited into the absorbing region per unit time and per unit volume. If an intensity modulated laser beam with intensity 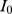 and modulation frequency 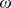 is used to deliver heat into the absorbing region, Eq. (1) reduces to its time independent form as,
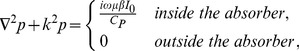
(2)where 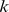 represents the wave number of the pressure wave and 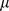 is the optical absorption coefficient of the absorbing medium. The PA field, generated by a spherical absorber, can easily be obtained by solving Eq. (2) and employing appropriate boundary conditions (i.e. the continuity of pressure and normal component of particle velocity at the spherical boundary). Thus, the solution of Eq. (2) becomes,
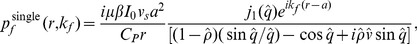
(3)where 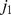 is the spherical Bessel function of order unity and 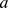 is the radius of the absorber. The argument of the spherical Bessel function 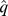 is related to the size of the object as 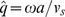. The dimensionless quantities, 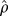 and 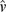 are defined as 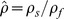 and 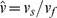 with 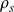 and 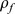 are the densities of the absorbing and fluid medium, respectively. The superscripts 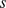 and 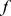 represent the absorbing and the surrounding medium, respectively. The notations 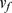 and 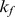 denote sound propagation speed and wave number of the acoustic wave within the surrounding fluid medium, respectively. The superscript single in Eq. (3) is used to state that the PA field is generated by a single source.

**Figure 3:**
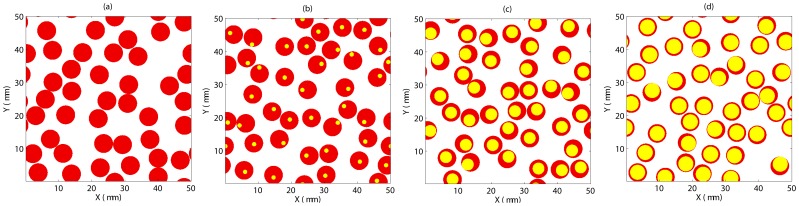
Simulated 2D tissue realizations with randomly placed non-overlapping RBCs under periodic boundary conditions. (a) Normal stage. (b) Ring stage. (c) Trophozoite stage. (d) Schizont stage. Each big circle in each figure represents a RBC and vacuoles are denoted by the inner circles. A 40% area fraction is occupied by the RBCs in each figure. As the time progresses, size of a vacuole increases and the quantity of hemoglobin in a cell decreases. For better visualization, 2D tissue configurations are shown but simulations have been carried out for 3D spatial distributions of cells.

**Table 1:** Estimated values of cellular optical absorption coefficient at different stages of infection.

Cell type	Cytoplasm		Vacuole		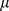 (m 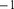 )	
	Volume ( 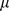 m^3^)	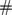 of Hb molecules (million)	Volume ( 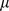 m^3^)	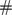 of Hmz molecules (million)	at 434 nm	at 700 nm
Normal	93.1	280	0.0	0	635159.21	2047.29
Ring	88.5	266	4.6	28	607530.78	4022.18
Trophozoite	57.5	173	35.6	214	423999.09	17141.06
Schizont	34.2	103	58.9	354	285856.95	27015.49

If the ROI contains a collection of spherical absorbers, the linear superposition principle can be applied to describe the resultant PA field and at a large distance 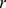 from the center of the ROI, it is given by [19], [20], [21],
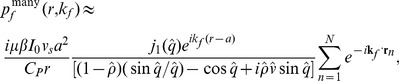
(4)where 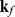 defines the direction of observation and 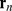 represents the position vector of the nth absorber. A schematic diagram illustrating the PA geometry is shown in Fig. 1. The superscript many states that an ensemble of PA sources is present within the ROI. In deriving Eq. (4), it has been assumed that the incident light intensity is same for all absorbers within the ROI. Therefore, attenuation of the light beam has not been considered in this derivation. The interaction between the pressure waves and the absorbers has also been ignored. Further, Eq. (4) is based on the single particle approach. It implies that fields from individual particles are summed up to obtain the resultant pressure field. The single particle approach works well for turbid medium and has been successfully utilized to explain experimental results in light and ultrasound scattering problems [24], [25].

**Figure 4:**
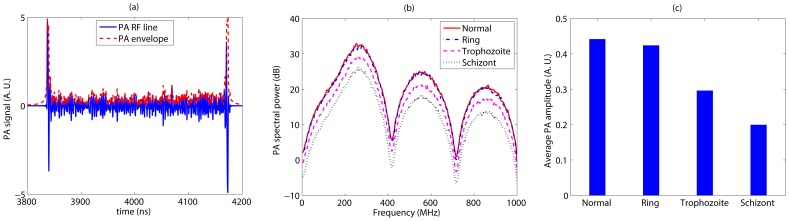
The NBL PA signal properties as a function of malarial infection for 434 nm incident illuminating radiation. (a) A representative wide bandwidth PA RF line computed from a 3D spatially random distribution of healthy RBCs. Associated signal envelope is also shown in this figure. (b) Plots of mean power spectrum for different samples. (c) The variation of mean signal amplitude as a function of intraerythrocytic stage of malarial parasite.

The time dependent pressure field can readily be obtained by taking the Fourier transformation of Eq. (4) and for a delta function input heating pulse it can be written as,
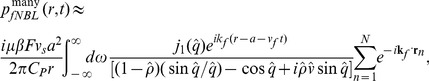
(5)where 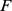 is the optical radiation fluence. Eq. (5) expresses an analytic signal containing all possible frequencies. The non-bandlimited nature of the signal is represented by the subscript NBL. For an analytic signal, the real part provides the PA RF signal and the imaginary part is the Hilbert transform of the real part [26].

In practice, the PA signals are detected by a receiver with finite bandwidth and thus, essentially converts a NBL signal into a bandlimited (BL) signal. The BL analytic signal can be obtained from 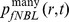 as [26],
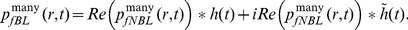
(6)where 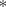 denotes the convolution operation and 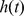 is the impulse response of the receiver. If a Gaussian function is used to model the frequency response profile of a transducer [27], one can write,
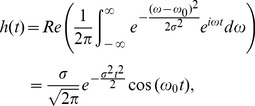
(7)and



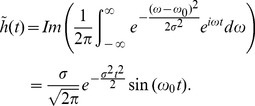
(8)Here, 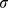 is related to the −6 dB bandwidth of the receiver. In this work, Eq. (6) has been computed to generate the BL signals for samples consisting of RBCs with different stages of malarial infection and compared with the corresponding NBL signals.

**Figure 5:**
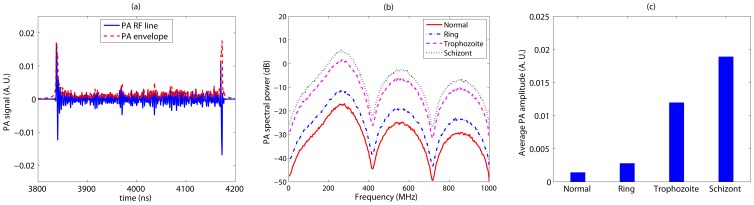
The variation of the NBL PA signal features with parasite growth when illuminated by 700 nm optical radiation. (a) A representative wide bandwidth PA RF line computed from a 3D spatially random distribution of normal RBCs. Associated signal envelope is also shown in this figure. (b) Plots of mean power spectrum for different samples. (c) The variation of mean signal amplitude with parasitic stage.

#### Evaluation of cellular Hmz concentration

There are two cores in a malaria infected RBC. The parasite vacuole acts as the inner core and the cytoplasmic region forms the outer core. It has been assumed that the Hmz molecules are homogeneously distributed within the inner core and the outer core contains the Hb molecules. For such a spherical absorber with two cores, the volume weighted cellular optical absorption coefficient can be cast as,
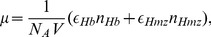
(9)where 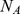 refers to Avogadro's number and 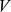 is the volume of a RBC. Here, 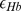 and 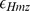 represent the molar extinction coefficients for the Hb and Hmz, respectively; 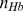 indicates how many Hb molecules are present within the cytoplasmic volume and 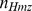 denotes the number of Hmz molecules that exist within the vacuole. There are two unknowns in Eq. (9) (i.e. 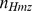 and 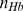). If the 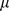 values are known at two wavelengths, one calculates,
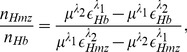
(10)where 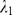 and 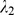 denote the incident optical wavelengths, respectively. Moreover, PA amplitude is linearly proportional to 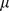 [see Eq. (4), Eq. (5)] and that yields,



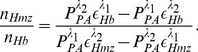
(11)Note that blood oxygen saturation has been assessed by a formula, which is analogous to Eq. (11) and sound results have been obtained [16], [20], [21]. Eq. (11) has been computed in this paper to examine how the ratio 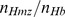 varies as the malarial parasite grows (both Hmz and Hb are attached to the same cell).

**Figure 6:**
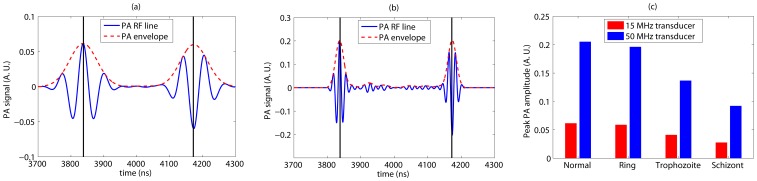
The BL PA signals for various receivers when probed with 434 nm laser beam. (a) Illustration of a bandlimited PA RF line along with its envelope computed from a 3D spatially random distribution of healthy RBCs. A transducer with 15 MHz as the center frequency and 60% bandwidth was used in this case. (b) Same as (a) but for a 50 MHz transducer. (c) The variation of peak signal amplitude as a function of parasitic stage for each transducer.

### Simulation Methods

#### Choice of the cellular mechanical and thermal properties

The volume of a cell was considered as 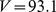 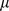
m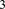 with radius, 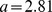 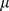
m [6]. The density and the speed of sound within the cell were taken as 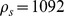 kg/m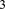 and 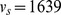 m/s, respectively [28]. The thermal expansion coefficient and the heat capacity per unit mass for an erythrocyte were assumed to be 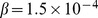 K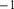 and 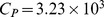 J kg
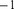
K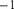, respectively [29]. The fluence of the incident optical radiation was fixed at 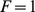 J m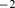, assuming uniform illumination of the cells within the ROI. The density and the speed of sound for the ambient medium (saline water) were chosen as 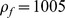 kg/m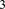 and 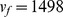 m/s, respectively [28].

**Figure 7:**
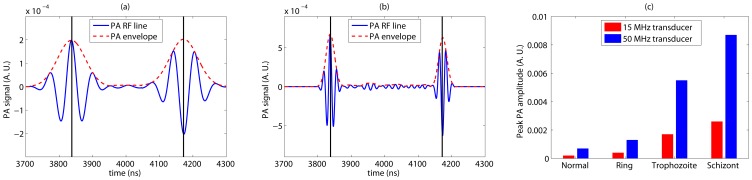
The BL PA signals for different transducers for 700 nm incident optical beam. (a) Illustrations of a bandlimited PA RF line and its envelope computed from a 3D spatially random distribution of healthy RBCs. A transducer with 15 MHz as the center frequency and 60% bandwidth was used in this case. (b) Same as (a) but for a 50 MHz transducer. (c) The variation of peak signal amplitude with the development of malarial parasites inside the host cells for each transducer.

#### Choice of the cellular optical absorption coefficient

Cellular optical absorption coefficient (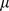) is another important parameter in PAs. It controls the PA signal amplitude emitted by a single cell [see Eq. (3)]. For a normal cell, 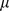 is determined by the concentration and oxygen saturation states of the constituent Hb molecules. However, for an infected cell, in addition to Hb molecules, Hmz molecules also play a role in defining the light absorption of that cell. In general, as the parasitic stage progresses inside the host RBC, contribution from the Hb molecules decreases but increases for the malaria pigment. Plots of molar extinction coefficients for the oxy-hemoglobin (HbO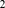), Hb and Hmz molecules are shown in Fig. 2. Spectral data for the HbO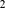 and Hb were taken from [30] and scaled up by a factor of 2.303 as discussed in [30]. Molar extinction coefficients at different optical wavelengths for the Hmz were obtained by dividing the absorbance data plotted in curve a, left panel of Fig. 1 of [9] with the molar concentration. Note that the molar extinction coefficient for the Hmz at 400 nm is about 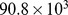 cm
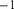
M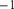 [11], and it was used to estimate the molar concentration. It is clear from Fig. 2 that molar extinction coefficients for the HbO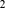, Hb and Hmz molecules do not differ greatly in the spectral range ∼450 to 582 nm. However, extinction coefficient curves are well separated outside this band expecting to provide significantly different cellular absorption coefficients for cells with various stages of malarial parasite than that of normal cells.

**Figure 8:**
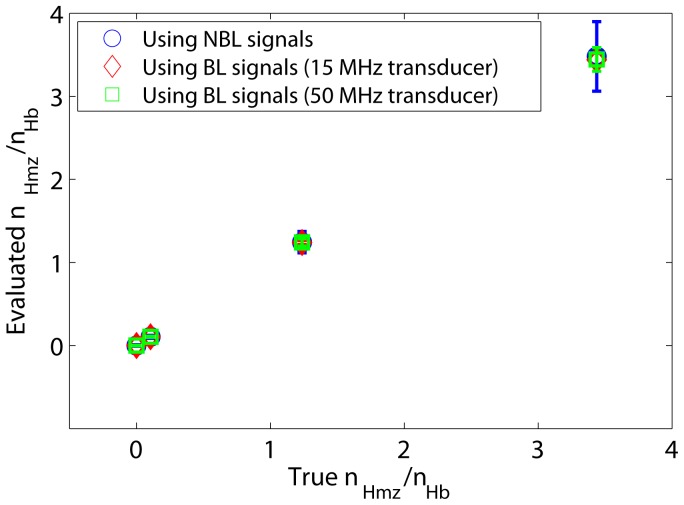
PA evaluation of number of intracellular Hmz molecules with respect to that of Hb.

The cellular absorption coefficients were estimated at 434 and 700 nm incident optical radiations by computing Eq. (9), that provided volume weighted absorption coefficient for each optical wavelength. The numerical values are presented in Table 1. In this work, it was assumed that normal as well as infected cells contained only Hb molecules at a concentration of 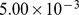 moles/l [31] and no HbO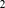 molecule was present. The Hmz molecules were assumed to be homogeneously deposited within the vacuoles for infected cells. As mentioned earlier, Hmz is structurally identical to 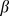-hematin, which is made of dimers of hematin molecules [7]. Thus, two iron atoms are present in a Hmz molecule, whereas each Hb molecule is composed of four iron ions. It is consequently expected that two Hmz molecules can be formed from each Hb molecule. In addition, in this work, we hypothesized that 100% of the haem released from the Hb molecules got converted into Hmz. This conversion rate is close to experimentally measured value [12]. In fact, approximately 95% of haem was found to be sequestered in Hmz [12]. The numerical values for the cytoplasmic volume containing the Hb molecules and volume of a vacuole packaging the Hmz molecules are presented in Table 1 for each type of cell. Associated numbers for the Hb and Hmz molecules are also provided in Table 1. These numerical values were estimated using the published experimental data [6]. Based on these values the 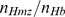 ratio for each infected stage was calculated. The parasitic stages are pictorially illustrated in Fig. 3. For better visualization, 2D spatially random distributions of RBCs are shown in Figs. 3(a)–(d) for those stages, respectively. It is clear from these figures that as the parasitic stage matures, size of a vacuole increases and cytoplasmic volume is reduced. It may further be mentioned that the area fraction occupied by a vacuole in a cell in each stage (as displayed in each figure) considered in the present study is equal to the experimentally measured volume fraction [6].

#### Construction of tissue realizations in 3D

The PA works involve detection of pressure waves by a transducer with a finite receiving bandwidth. The sensitivity of the transducer is generally maximum at its center frequency. The wavelengths associated with the detected waves are generally much greater than the size of a RBC and thus, the effect of shape on angle dependent PA amplitude can be ignored. Additionally, for biconcave shape, it is not possible to obtain a closed form solution of Eq. (2). Therefore, in this study, each erythrocyte was approximated as a fluid sphere and the PA signals were simulated for a collection of RBCs. Note that RBCs are the most numerous corpuscles in blood (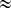
98% particles are the RBCs). Further, RBCs contain Hb molecules which are the dominant chromophores for the portion of optical spectrum used in the PA studies, allow cells to absorb light and emit PA waves [17]. Hence, the contributions from the white blood cells and platelets generating PA waves were neglected in this study.

Simulations using 2D spatial organizations of RBCs were conducted by us in recent PA studies [19], [20], [21]. In the context of ultrasound backscattering by cells, both 2D and 3D simulations were performed by us [32], [33]. 2D simulations are computationally less expensive than that of 3D simulations and capable to provide meaningful results. However, 3D configurations better approximate experiments. That is why an attempt has been made here to generate 3D tissue samples and consequently, to simulate the PA signals from such samples. The size of the ROI was chosen as 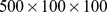 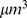. This volume was populated with spheres (i.e. RBCs) occupying a packing fraction of 40%. A 40% packing fraction was preferred because it is close to normal level of hematocrit (defined as the volume fraction occupied by the RBCs in blood) in human blood [25]. The optical radiation propagated along the longer dimension. This allowed the emitted acoustic waves, with wavelengths shorter than that length, to interfere both constructively and destructively in the forward (along the direction of light propagation) and backward directions. The PA signals in this study were computed in the backward direction.

A Monte Carlo algorithm, referred to as the random sequential adsorption (RSA) technique, could be used to generate spatial distributions of spheres in 3D [34]. During the RSA procedure, the particles are randomly and irreversibly adsorbed within the ROI maintaining the condition that they do not overlap with the existing particles. This technique can generate random loose pack configurations for spheres with equal radii and the attainable packing fraction is 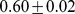 in 3D [34]. However, computation time in this technique increases almost exponentially as the number of particles increases. Further, as it is an irreversible procedure, sometimes it does not converge particularly at higher concentrations of particles [34]. Therefore, in this study, the ROI was divided into a number of sub-blocks with a size of 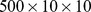 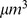 for each. Each sub-block was filled with the particles separately using the RSA approach. For the particles situating at the boundaries, the non-overlapping condition was also checked with that of the neighboring sub-blocks. If the RSA technique did not provide a possible configuration for a sub-block within a suitable time limit, generated coordinates were deleted and the procedure was repeated from the scratch. A maximum 7 such attempts were made to generate a possible configuration for that sub-block. If it was not sufficient, a modified RSA approach was applied. In this approach, 30% of the particles (arbitrarily chosen) were placed first within a sub-block employing the RSA. After that, each particle from the remaining 70% particles were positioned on the surface of a existing particle (randomly chosen) under the non-overlapping condition. It was observed that this approach generated random positions of RBCs faster than the RSA. It may further be noted that it is the building block of various algorithms that have been developed and implemented to achieve random close packing of equal spheres providing a packing fraction of 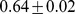 [35], [36].

Some representative tissue configurations with non-overlapping randomly placed cells are shown in Fig. 3. Although, 2D realizations are presented for better visualization of the spatial organizations of cells but signals were simulated from 3D samples.

#### PA signal generation

Once the random positioning of cells in 3D were completed, the integration in Eq. (5) was evaluated numerically using the trapezoidal rule to simulate wide bandwidth PA signals. The contributions from a wide range of frequencies (0.001 to 1000 MHz with a resolution of 0.25 MHz) were summed up. The time series pressure data were generated at a sampling frequency of 4 GHz. The real part of the time series data provided the RF signal. The signal amplitude at each time point was obtained from the square root of the sum of squares of the real and imaginary parts. The PA signals were generated for four different types of samples associated with normal, ring, trophozoite and schizont stages. A computer code was written in C and ran on a IBM server (OS: Red Hat Enterprise Linux 5.3EL, Processor: Intel Xeon 3 GHz, RAM: 8 GB, Quad Core, 32-bit). For each case, 200 RF lines were obtained from 200 different tissue configurations and the required computer execution time was about 13 hrs. The simulated data were then imported in MATLAB R2009b for post processing. The average PA spectral power and signal amplitude were obtained for each incident optical beam to investigate the effect of malarial parasite on the PA signals.

The simulated wide bandwidth signals were convolved with the impulse response of a receiver to generate bandlimited signals [see Eq. (6)]. Two receiving transducers with 15 and 50 MHz as the center frequencies were studied in this work. These receivers are on the higher side compared to the diagnostic ultrasound frequency range (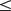
10 MHz). A 60% bandwidth was considered for each case. In these cases also PA signal amplitudes were computed to examine as to how bandlimited signal properties vary with time course of malarial parasite.

## Results

Fig. 4(a) displays a wide bandwidth PA RF line when simulated from a 3D tissue sample with spatially random distribution of healthy RBCs and illuminated by 434 nm optical radiation. Associated signal envelope is also plotted in this figure. It can be seen that a strong PA signal is obtained from each boundary, where signals from various cells add up coherently. However, signal amplitude corresponding to the central region is reduced significantly compared to those of the boundaries due to incoherent addition of acoustic waves from randomly located RBCs. The mean power spectra for different stages are plotted in Fig. 4(b) over a wade range of ultrasound frequencies (MHz to GHz). Fig. 4(b) demonstrates that the ultrasound spectral power decreases as the parasitic stage progresses over the entire frequency range. Further, spectral lines corresponding to normal and ring stages are not separable because the difference of cellular optical absorption coefficient between these two stages is negligible for this incident light beam. However, other lines are distinguishably separable. For example, spectral power at about 7.5 MHz for the schizont stage is nearly 6 dB less than that of the normal cells. Fig. 4(c) illustrates that the PA signal amplitude decreases as the parasite matures. As expected, signal amplitudes for normal and ring stages are comparable but others differ significantly.

Fig. 5(a) demonstrates a wide bandwidth PA RF line along with its envelope when computed from a 3D tissue sample with randomly positioned healthy RBCs and irradiated with 700 nm optical beam. As in the previous case, signal amplitudes are maximal at the boundaries. Nevertheless, signal strength at this optical wavelength is weaker than that of the other light beam. This is because light absorption by a cell at 700 nm is less than that of 434 nm (see Table 1). Plots of ultrasound mean power spectrum are shown in Fig. 5(b) corresponding to various intraerythrocytic stages of malarial parasite. The spectral power increases as the parasite stage proceeds at any ultrasound frequency. The spectral power at nearly 7.5 MHz differs by 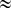
6 dB between the normal and ring stages, whereas, it is about 22 dB between the normal and schizont stages at the same ultrasound frequency. An increasing trend of the PA amplitude with increasing infection has been observed and presented in Fig. 5(c).

A representative plot of a bandlimited signal generated by convolving a NBL signal for normal cells as presented in Fig. 4(a) with the Gaussian function modeling a 15 MHz transducer is shown in Fig. 6(a). The signal envelope has also been outlined in Fig. 6(a). It demonstrates that the random variation of amplitude is greatly reduced than that of the NBL signal [see Fig. 4(a)]. This can be linked to the fact that the Gaussian function filters out high frequency components associated with the NBL signal leading to a smooth signal. The signal amplitudes at the boundaries are much stronger than that of the central region which have been observed as well in case of the NBL signals [see Fig. 4(a)]. However, the signals at the boundaries are out of phase by 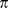 radians and it originates from the nature of thermoelastic expansion [21]. In Fig. 6(b), the effect of 50 MHz transducer on a NBL signal is depicted. Signal strength in this case is higher than that of Fig. 6(a), because the PA amplitude at 50 MHz is greater compared to that of 15 MHz [see Fig. 4(b)]. In addition, slightly more random fluctuation of amplitude is noticed in this case due to the presence of high frequency components in comparison to Fig. 6(a). The PA signal amplitude as a function of parasitic stage is plotted in Fig. 6(c) for each receiver. It shows that the signal amplitude decreases as the parasites evolve inside the host RBCs. The trends are identical for both the cases.

A simulated bandlimited signal computed by convolving a NBL signal with the Gaussian function mimicking a 15 MHz transducer is plotted in Fig. 7(a). The corresponding NBL signal is shown in Fig. 5(a). In Fig. 7(b), output of a 50 MHz transducer is presented. Fig. 7(c) plots the PA amplitude as a function of intraerythrocytic stage of malarial parasite for each transducer. Signal properties are similar to that of Fig. 6. However, in this case the PA amplitude increases as the parasites grow within the RBCs.

The accuracy of the PA technique to evaluate the presence and quantity of Hmz molecules in a cell has also been assessed in this study using simulated RF lines generated at 434 and 700 nm optical wavelengths and computing Eq. (11). Both the NBL and the BL signals have been utilized for this purpose. Fig. 8 plots the evaluated 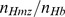 (mean 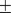 SD) as a function of true 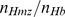. Note that the true 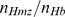 was known for each stage and it became fixed during the computation of 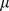 for each case (see Table 1). This figure reveals that estimated values are in excellent agreement with those of the actual values for the NBL and the BL cases considered in this study.

## Discussion

The effect of membrane stiffness on the PA signals has been ignored, although it under goes significant alterations as the parasites grow inside the host RBCs [6]. This assumption is based on the fact that mechanical contrast does not have a major role in the PAs while optical contrast plays the dominant role. In this work, the oxygen saturation level for each RBC in a blood sample (either normal or infected) was considered to be 0%. However, the oxygen saturation of an erythrocyte may vary from 0 to 100% and in that case, HbO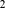 would also contribute to the cellular absorption coefficient. The effect of blood oxygenation could easily be incorporated by introducing an additional term for HbO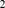 in Eq. (9).

Additionally, we assumed that Hmz molecules, produced due to the digestion of Hb molecules by the parasites, were homogeneously deposited inside the vacuoles. Based on this assumption, we estimated that the cellular absorption coefficient *vis-a-vis* the PA signal amplitude was shown to greatly vary for certain incident laser radiations during the intraerythrocytic development of malarial parasite. Nevertheless, it is known that the parasitic vacuoles contain localized Hmz crystals [6]. It is not clear yet whether there would be at all any difference in light absorption between these cases and so further investigation is required to resolve this issue.

It is note worthy to mention that at the mature states of parasites, the infected cells release Hmz crystals due to hemolysis. It is intuitively expected that blood plasma in that case will absorb light and consequently produce the PA signals. However, in this model, we assumed that the PA signals were generated by the cells only. Therefore, it would be interesting in future to include this effect within the theoretical model and study how the background signal generated by the surrounding fluid medium would modify the overall PA signal properties.

This article deals with a detailed description of a Monte Carlo algorithm for simulating tissue configurations in 3D. The entire ROI was divided into a number of equal-sized sub-blocks and each sub-block was populated separately through non-overlapping spheres mimicking RBCs. This algorithm allowed us to generate sufficiently big tissue realizations. Nevertheless, this procedure could easily be modified to facilitate parallel computing and in that case even bigger ROI could be simulated within a realistic time limit.

The simulation results show that the differences in the PA spectral power are about −6 dB and 22 dB between normal blood and infected blood with RBCs at the schizont stage for 434 and 700 nm incident optical radiations, respectively. These numerical values are quite high indicating that the differentiation of intraerythrocytic stages should be feasible in practice using the PA technique. However, an experimental verification is required to support this theoretical prediction and also to validate the assumptions made in this study. Future work warrants to conduct experiments with malaria infected cells and assess the potential of the PA technique to differentiate intraerythrocytic stages of malarial parasite.

### Conclusions

A computer simulation study examining the potential of the PA technique to differentiate various stages of malarial infection in human blood is presented. For this purpose, a recently developed frequency domain theoretical model was used. The fundamental assumption has been that the malarial parasite during its development inside the host RBC causes major changes in cellular optical absorption (as it converts significant amount of Hb into Hmz) and thus, induces alterations in the PA emission of a cell. The PA signals from the blood samples corresponding to ring, trophozoite and schizont stages were simulated and compared with normal blood composed of healthy cells. The analysis of wide bandwidth signals consisting of frequencies ranging from MHz to GHz reveals that as the parasites grow inside the RBCs, the PA amplitude decreases and increases for 434 and 700 nm incident radiations, respectively. The signal amplitude for the blood sample with RBCs at the schizont stage was estimated to be decreased and increased nearly by a factor of 2 and 13 than those of the normal blood for the same incident lasers, respectively. Corresponding PA spectral power at 7.5 MHz was computed to be reduced by 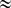
6 dB and enhanced by 22 dB, respectively. The bandlimited signals were obtained by convolving the wide bandwidth signals with a Gaussian function modeling the impulse response of a transducer. Two transducers with 15 and 50 MHz as the center frequencies were studied. Bandlimited signal characteristics were observed to be similar to that of wide bandwidth signals for each case. The presence and amount of Hmz in RBCs were examined using the simulated PA signals and it was found that quite accurate assessment of Hmz is possible employing two wavelengths PA system. It can be concluded that the PA technique may have the ability to differentiate intraerythrocytic stages of malarial parasite if an appropriate optical wavelength is used to illuminate the blood samples.
